# Case report: Cystic hygroma accompanied with campomelic dysplasia in the first trimester caused by haploinsufficiency with *SOX9* deletion

**DOI:** 10.3389/fgene.2022.950271

**Published:** 2022-08-29

**Authors:** Xijing Liu, Jianmin Wang, Mei Yang, Tian Tian, Ting Hu

**Affiliations:** ^1^ Department of Medical Genetics, West China Second University Hospital, Sichuan University, Chengdu, China; ^2^ Department of Obstetrics and Gynecology, West China Second University Hospital, Sichuan University, Chengdu, China; ^3^ Key Laboratory of Birth Defects and Related Diseases of Women and Children (Sichuan University), Ministry of Education, Chengdu, China; ^4^ Department of Diagnostic Ultrasound, West China Second University Hospital, Sichuan University, Chengdu, China

**Keywords:** campomelic dysplasia, cystic hygroma, SOX9, ultrasound, chromosomal microarray analysis

## Abstract

**Introduction:** Campomelic dysplasia (CD) is a rare autosomal dominant skeletal malformation syndrome characterized by shortness and bowing of the lower extremities with or without XY sex reversal. Diagnosis using ultrasonography is most often made in the latter half of pregnancy. Intragenic heterozygous mutations in SOX9 are responsible for most cases of CD. CD caused by SOX9 deletion is a rare condition.

**Case presentation:** We present a single case report of an individual with cystic hygroma accompanied by CD, which was detected by ultrasound in the first trimester. Chromosomal microarray analysis (CMA) was performed to determine copy number variants, whereas whole exome sequencing (WES) was performed to elucidate single-nucleotide variants. Chorionic villus sampling was performed to enable such analyses. Ultimately, CMA detected a 606 kb deletion in the 17q24.3 region with only one protein-coding gene (SOX9). However, no mutation in the SOX9 protein-coding sequence was detected by WES.

**Conclusion:** When cystic hygroma is detected, prenatal diagnoses for skeletal dysplasia by ultrasound are likely to be confirmed in the first trimester. We propose a comprehensive prenatal diagnostic strategy that combines CMA and WES to diagnose fetuses with cystic hygroma accompanied by skeletal dysplasia.

## Introduction

Campomelic dysplasia (CD, MIM: #114290) is a rare autosomal dominant skeletal malformation syndrome characterized by an under-mineralized skeleton with shortness and bowing of lower extremities and hypoplasia of scapular and pelvic bones. Moreover, other notable anatomical characteristics include a small chest, 11 pairs of ribs, clubfeet, a cleft palate, and micrognathia (Mansour et al., 1995; Jain and Sen, 2014). Two-thirds of affected males showed XY sex reversal or a lesser degree of genital defects (Mansour et al., 1995). CD is a semi-lethal disorder because most patients die during the neonatal period secondary to respiratory distress (Mansour et al., 2002).

*SOX9* (SRY-related HMG-box gene 9, OMIM:608160), located in chromosome 17q24.3, is a transcription factor that plays a critical role in the development of skeletal and reproductive systems by inducing chondrocyte differentiation and anti-Mullerian hormone expression (Wagner et al., 1994; Olney et al., 1999). Most cases of CD are caused by intragenic heterozygous mutations in *SOX9*, including missense, nonsense, frameshift, and splice mutations (Unger et al., 1993). A second mechanism for CD is balanced chromosomal rearrangements, including translocations, inversions, and deletions, which do not affect *SOX9* but cause interruptions upstream or downstream of *SOX9* (Unger et al., 1993; Gordon et al., 2009; Lecointre et al., 2009). Additionally, a few CDs are caused by large deletions covering *SOX9* (Olney et al., 1999; Pop et al., 2004; Smyk et al., 2007; Kayhan et al., 2019).

CD might be suspected after shortened lower limbs and femoral angulation are detected by prenatal ultrasonography, although observations are often made in the latter half of pregnancy. Thus, antenatal diagnosis of CD is difficult during the first trimester (Tongsong et al., 2000). We report an individual with cystic hygroma accompanied by CD in the first trimester and confirm that such a phenotype resulted from *SOX9* deletion.

## Case description

**FIGURE 1:**
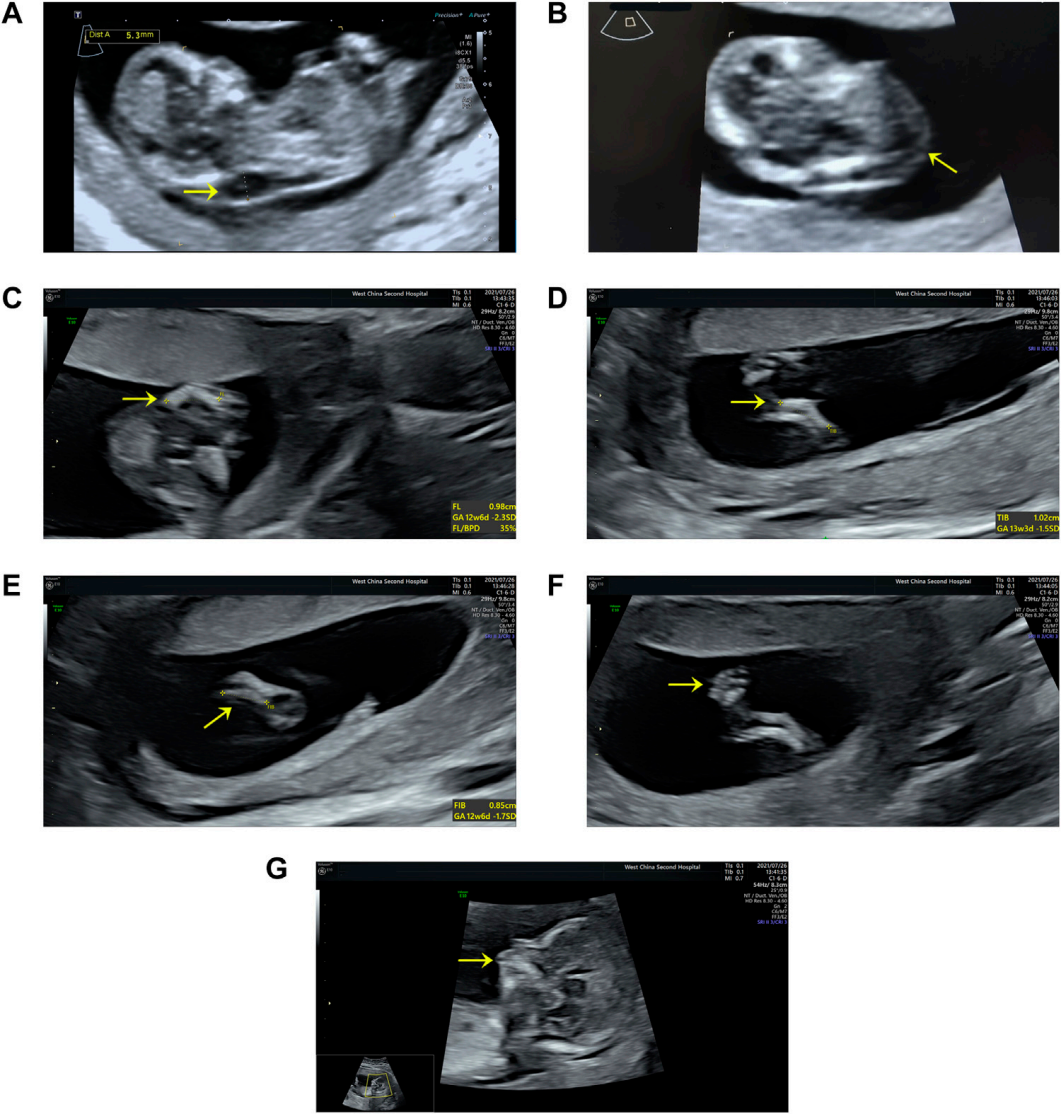
Ultrasound findings: **(A)** Cystic hygroma measuring 18 × 6 × 14 mm (CRL:60.9 mm) at the mid-sagittal plane. **(B)** Cystic hygroma in a transverse view. **(C)** Short and bowed femurs (<first centile). **(D)** Sagittal anterior angulated tibiae (fifth centile). **(E)** Sagittal anterior angulated fibula (fifth centile). **(F)** Unilateral equinovarus clubfoot. **(G)** Micrognathia.

A 27-year-old nulliparous woman was referred to the Prenatal Diagnosis Center of West China Second University Hospital of Sichuan University (a tertiary referral center) for consultation and sonography concerning a cystic hygroma discovered at 13 weeks and 4 days. The crown-rump length was 60.9 mm and a cystic hygroma measuring 18 × 6 × 14 mm was confirmed (Figures 1A,B). The couple has no history of consanguinity. At 14 weeks and 6 days, a detailed fetal anomaly scan revealed severe short and bowed femurs (<first centile), sagittal anterior-angulated tibiae and fibula (fifth centile), unilateral equinovarus clubfoot, and micrognathia (Figures 1C–G). The biparietal diameter was 27.9 mm, while the thorax and upper limbs appeared normal. The fetus was found to have female external genitalia. A presumptive diagnosis of skeletal dysplasia was made. However, a precise diagnosis was difficult to achieve based on limited ultrasound findings. Chorionic villus sampling was performed to enable chromosomal microarray analysis (CMA) and whole exome sequencing (WES).

The study was approved by the Medical Ethics Committee of the West China Second University Hospital of Sichuan University in China.

## Diagnostic assessment

### Methods

#### Chromosomal microarray analysis

We conducted the chromosomal microarray analysis using CytoScan 750 K Array (Affymetrix, Santa Clara, CA, United States). The samples were prepared according to the manufacturer’s instructions. The array scan data were visualized using the Chromosome Analysis Suite v4.1 software. The GRCh38 genome was used for annotation. The pathogenicity of copy number variants (CNVs) was based on the technical standards of the American College of Medical Genetics and Genomics (ACMG) and the Clinical Genome Resource (ClinGen) (Riggs et al., 2020).

#### Whole exome sequencing

The Nano WES Human Exome V1 (Berry Genomics, Beijing, China) was used to capture the sequences. The enriched library was sequenced on a NovaSeq 6,000 with 150 paired-end reads. The reads were mapped to a human reference genome (GRCh38) using BWA (v0.7.15). Variant calling was performed using the Verita Trekker (v1.2.0.2). After filtering the variants with the classic population frequency databases, we rated the pathogenicity of the remaining mutations according to the ACMG guidelines, as previously described (Chen et al., 2022).

## Methods

**FIGURE 2:**
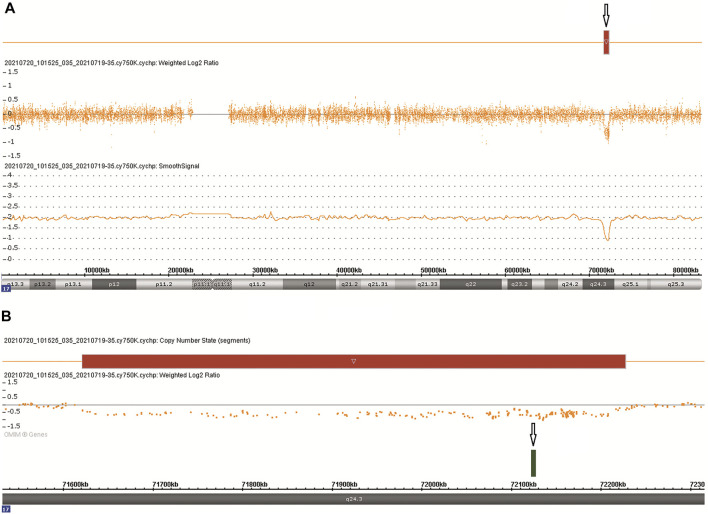
Chromosomal microarray findings: **(A)** A 606 kb deletion in the 17q24.3 region. **(B)** The deletion contains one OMIM gene SOX9.

CMA was performed following chorionic villus sampling, which revealed a 606 kb deletion [arr [GRCh38] 17q24.3 (71,620,225_72,226,459)x1] in the 17q24.3 region (Figure 2A). The deletion contained one protein-coding gene (*SOX9*) and five other genes (*LINC01152*, *LINC02097*, *MYL6P5*, *ROCR,* and *SOX9-AS1*), which extended from 501 kb upstream to 100 kb downstream of *SOX9* (Figure 2B). The deletion overlapped only 9.3% of the *SOX9* upstream enhancer region, which is identified as a haploinsufficiency (HI) genomic region (ClinGen database (http://www.ncbi.nlm.nih.gov/projects/dbvar/clingen/)). The sex chromosome of the fetus was XX. This deletion was not detected in the couple, suggesting that a *de novo* event had occurred. The CNVs were classified as “Pathogenic” according to ACMG and ClinGen Technical standards (Riggs et al., 2020). Sequentially, a trio-WES was performed for genetic factors of skeletal dysplasia in addition to *SOX9* deletion, and yet the analysis yielded negative results.

The diagnosis of CD was confirmed based on the SNP-array results and fetal ultrasound findings.

## Discussion

We report a case of CD with cystic hygroma detected in the first trimester by ultrasonography, and in which further testing confirmed the presence of *SOX9* deletion. Commonly, prenatal diagnosis of skeletal dysplasia is performed by ultrasonography during the second trimester. Recently, Kayhan et al. (2019) reported a 46, XY prenatal case of CD with *SOX9* deletion, characterized by micrognathia, bowed limbs, clubfeet, and female genitalia in the second trimester. However, ultrasound in the first trimester appeared normal, and no apparent anomalies, such as cystic hygroma or increased nuchal translucency, were observed. However, a cystic hygroma or increased nuchal translucency during the first 3 months has been reported to be a relatively frequent sign in fetuses subsequently diagnosed with skeletal dysplasia (Massardier et al., 2008; Zhen et al., 2015). As in our case, a detailed fetal anomaly scan was performed sequentially when cystic hygroma was detected, and skeletal dysplasia was confirmed. Kenkhuis et al. (2018) concluded that it is possible to detect approximately half of the prenatally detectable structural anomalies with an early scan performed in the first trimester by competent fetal sonographers. Thus, a detailed ultrasonography should be immediately performed when cystic hygroma or increased nuchal translucency is detected in the first trimester.

CD is an autosomal dominant disease usually caused by a heterozygous pathogenic variant of *SOX9* (Unger et al., 1993)*.* Most CD cases show heterozygous *de novo* mutations in the coding region of *SOX9*. A small number of cases have a heterozygous interstitial deletion or reciprocal translocation of 17q24.3-q25.1, which involves *SOX9* or its regulatory region (Unger et al., 1993; Gordon et al., 2009; Lecointre et al., 2009). Currently, the haploinsufficiency of *SOX9* is disputable. In the last few decades, loss-of-function mutations in one of the three *SOX9* exons have been identified in CD. Therefore, it has been assumed that the disease results from the haploinsufficiency of *SOX9* (Unger et al., 1993; Ninomiya et al., 2000). However, the research conducted by Csukasi et al. (2019) suggested a dominant-negative nature to *SOX9* mutations in CD. As isolated *SOX9* deletion is rare, the evidence for *SOX9* haploinsufficiency is insufficient.

Currently, only four CD cases have been reported with the complete deletion of the *SOX9* gene (Olney et al., 1999; Pop et al., 2004; Smyk et al., 2007; Kayhan et al., 2019). Olney et al. (1999) first reported an infant with CD who has an interstitial deletion from 17q23.3 to q24.3 by karyotyping, whereas Pop et al. (2004) reported a male CD patient with a *de novo* deletion of at least 4.0 Mb, and Smyk et al. (2007) reported a female CD patient with a paternally inherited deletion of ∼4.7 Mb in size. Interestingly, all the three cases completely overlapped the *SOX9* upstream enhancer region that is positioned 2 Mb 5’ to *SOX9,* and which is associated with the Pierre Robin sequence (PRS, MIM #261800). PRS, a partial phenotype of CD, is characterized by micrognathia, cleft palate, and glossoptosis (Lecointre et al., 2009). Thus, the evidence of haploinsufficiency in *SOX9* deletion-induced CD remains elusive. Kayhan et al. (2019) performed a prenatal array comparative genomic hybridization on a 46, XY CD fetus and revealed an 886 kb deletion in the 17q24.3 region, which included the entire *SOX9* gene. Although the deletion also overlapped approximately 29.3% of the *SOX9* upstream enhancer region, it is difficult to confirm that *SOX9* deletion is solely responsible for the CD phenotype.

The genotype–phenotype correlation of *SOX9* remains unclear. Although loss-of-function mutations have been identified in some CD cases, functional studies suggest a dominant-negative phenotype (Wagner et al., 1994; Ninomiya et al., 2000; Jain and Sen, 2014; Csukasi et al., 2019). The advent of CMA has revealed CNVs, including both the coding and regulatory regions of *SOX9* (Croft et al., 2018). The utility of WES elucidated single-nucleotide variants of genetic disorders that were not detectable by CMA. However, deletions or duplications associated with cis-regulatory elements upstream of *SOX9* were not detected by the algorithms (Mansour et al., 2002). Therefore, we performed CMA combined with WES as an unbiased approach when cystic hygroma and skeletal dysplasia were detected. In our case, the phenotype of the fetus revealed by ultrasonography was consistent with the loss of *SOX9*, providing an increasing evidence of *SOX9* haploinsufficiency*.*

With the advancements in molecular detection technology, the prenatal diagnosis of fetuses at risk for genetic disorders has rapidly increased in recent years. The choice of application of CMA and WES deserves consideration. When a fetal anomaly is observed, we support the recommendations released by the International Society for Prenatal Diagnosis (Van den Veyver et al., 2022), which contends that, if no genetic diagnosis is found after CMA, a fetus with a major single anomaly or multiple organ system anomalies will benefit from WES or other genome sequencing methods. If the anomaly “pattern” strongly suggests a single genetic disorder with no prior genetic testing, CMA should be run before or parallel to WES. In this case, a cystic hygroma was first observed, and CMA was the prior genetic test. After a detailed ultrasonography, CD was highly suspected, and whether WES could have been a prior genetic test is worth considering, as CD is a disorder mainly caused by *SOX9* heterozygous *de novo* mutations.

In clinical practice, when a cystic hygroma or increased nuchal translucency is detected using a detailed ultrasonography, prenatal diagnoses for skeletal dysplasia are likely to be confirmed in the first trimester. Since a meta-analysis has found that the highest exome sequencing diagnostic yields occurred in fetuses with skeletal abnormalities (53% [95% CI 42–63%], *p* < 0.0001) (Mellis et al., 2022), the prenatal diagnostic strategy for fetuses with cystic hygroma accompanied by skeletal dysplasia requires more clinical experience.

### Patient perspective

After receiving the genetic and ultrasound reports, diagnosis was established, and the couple decided to terminate the pregnancy. The couple provided written informed consent to participate in this study. Written informed consent was obtained from each individual for the publication of potentially identifiable images or data included in this article.

## Data Availability

The datasets presented in this study can be found in online repositories. The names of the repository/repositories and accession number(s) can be found in the article.
